# Endotoxin-induced acute lung injury in mice with postnatal deletion of nephronectin

**DOI:** 10.1371/journal.pone.0268398

**Published:** 2022-05-12

**Authors:** Carole L. Wilson, Chi F. Hung, Lynn M. Schnapp

**Affiliations:** 1 Division of Pulmonary, Critical Care, Allergy, Sleep Medicine, Dept of Medicine, School of Medicine and Public Health, University of Wisconsin-Madison, Madison, Wisconsin, United States of America; 2 Division of Pulmonary, Critical Care, Allergy and Sleep Medicine, University of Washington, Seattle, Washington, United States of America; Children’s Hospital of Los Angeles, UNITED STATES

## Abstract

Acute injury of the lung involves damage to the epithelium and its underlying extracellular matrix (ECM), the basement membrane (BM). How BMs contribute to injury resolution is poorly understood. Nephronectin (NPNT) is a high-affinity ligand for integrin α8β1 and, although first identified in the mouse kidney, is prominently expressed in the lung, where it localizes to BMs in the alveoli. To determine if NPNT plays a role in acute injury and inflammation of the lung, we developed a model for postnatal deletion of NPNT using mice with a floxed allele of *Npnt* in combination with a tamoxifen-inducible Cre recombinase expressed at the ROSA locus. Expression of NPNT was substantially reduced in lungs from tamoxifen-treated Cre+ animals. Cre+ mice and Cre- controls were given *E*. *coli* LPS by oropharyngeal aspiration to induce injury and inflammation. In Cre- lungs, although both *Npnt* and *Itga8* (integrin α8) transcripts were downregulated at the peak of inflammation, NPNT protein was still detectable. While the onset of inflammation was similar for Cre+ and Cre-, NPNT-deficient lungs still had thickened alveolar septa and there were increased macrophages in the bronchoalveolar lavage fluid (BALF) in the resolution phase. BALF from Cre+ lungs was more chemotactic for bone marrow-derived macrophages than Cre- in *in vitro* experiments, but there were no differences in the elaboration of chemokines *in vivo*. We speculate that absence of NPNT in BMs of the alveoli impairs or delays inflammatory and injury resolution in this model, but further studies are needed to establish the precise role of NPNT in tissue repair.

## Introduction

Basement membranes (BMs) in the lung are an integral part of the air-blood barrier. During acute lung injury, there is localized disruption of the BM, leading to breaching of the air-blood barrier and extravasation of leukocytes into the alveolar space. Resolution of inflammation must occur for re-establishment of tissue homeostasis (reviewed in [1, 2]). In addition to the main structural proteins, collagen IV and laminin, the lung BM contains proteoglycans and non-structural proteins, including nephronectin (NPNT). NPNT, also known as POEM (for preosteoblast EGF-like repeat protein with MAM domain) [3], is a secreted protein that becomes incorporated into the BM in several organs, including kidney, skin, and lung in mice and humans [4–7], and is proposed to function in development and tissue homeostasis in these organs [4, 7]. NPNT was first identified in the mouse embryonic kidney as the key ligand for integrin α8β1 in mediating nephron morphogenesis [5]. Mice with global deletion of either NPNT or integrin α8 exhibit partial or complete kidney agenesis or hypoplasia because of delayed invasion of the mesenteric mesenchyme by the ureteric bud and insufficient branching of the bud [8, 9].

Previous work has implicated NPNT in tissue injury, inflammation, and repair. For example, NPNT is upregulated in mesenchymal cells of the liver in mouse models of acute and chronic hepatitis, where the protein is involved in the recruitment of CD4-positive cells through its RGD and synergy sites [10]. From proteomic studies, NPNT emerged as a protein that is regulated in bleomycin-induced inflammation and fibrosis in mice [11, 12]. During experimental renal injury, NPNT is expressed by tubule cells early in the regeneration process [13] and may be a candidate biomarker for glomerular repair in human nephrotic syndrome [14]. In human lung, silicosis is associated with increased serum levels of soluble NPNT [15]. Genome-wide association studies identified *NPNT* as part of a locus containing single-nucleotide polymorphisms that correlate with measures of lung function [16, 17]. A splicing variant of *NPNT* has been linked to chronic obstructive pulmonary disease [18].

In the adult mouse, the lung has the highest expression of NPNT of all the organs that have been examined [19]. *Npnt* mRNA is highly expressed in alveolar fibroblasts as well as epithelial cells [20, 21]. Whether NPNT is involved in lung injury and resolution is not known. To define the role of NPNT in acute lung injury, we generated mice with conditional deletion of NPNT in all cells and subjected these animals, along with NPNT-replete controls, to an LPS model of lung injury and inflammation.

## Materials and methods

### Mice

The strain carrying a floxed allele of *Npnt* (Npnt-Flox) in which exon 1 is flanked by loxP sites (B6;129P2-*Npnt*^*tm1Lfr*^/Mmmh) [9] was obtained from Mutant Mouse Regional Resource Center U42OD010918 and backcrossed for 10 generations onto the C57BL/6J background. For postnatal deletion of NPNT, the ROSA-CreERT2 (strain #008463; Jackson Labs) allele (Gt(ROSA)26Sor^*tm1(cre/ERT2)Tyj*^) was combined with the Npnt-Flox allele by breeding. Pups from crosses of Npnt-Flox/Flox with ROSA-CreERT2/+;Npnt-Flox/Flox were weaned and maintained on a diet containing tamoxifen (600 mg tamoxifen citrate/kg pellets; Envigo) for 3–4 weeks, with 2–9 weeks of wash-out on normal chow. Both Cre- and Cre+ pups were given the tamoxifen diet. To identify pups inheriting the CreERT2 allele, genotyping of tail biopsy genomic DNA was performed with the following PCR primers in a single reaction: 5’-AAAGTCGCTCTGAGTTGTTAT-3’, 5’-GGAGCGGGAGAAATGGATATG-3’, and 5’-CCTGATCCTGGCAATTTCG-3’. Presence of the floxed allele and its conversion to the knockout (KO) allele by tamoxifen exposure were assessed by genotyping tail biopsy genomic DNA using the following primers: 5’-GCAACCTTCAGCGTCCC-3’ and 5’-CAGTCCATCCTGATCACTACTGGCTGTA-3’ for Npnt-Flox and 5’-CAGAACGCGTACTTCCACTTCC-3’ and 5’-CAGTCCATCCTGATCACTACTGGCTGTA-3’ for Npnt KO.

For harvesting NPNT KO bone marrow, mice with global deletion of NPNT were generated by crossing the Npnt-Flox allele into the Pdgfrb-Cre transgenic [22] (a gift from Dr. Dean Sheppard at UCSF), which exhibits sporadic germline activity in Cre+ dams. PCR genotyping of tail biopsy genomic DNA was used to identify progeny that had the *Npnt* null allele without inheritance of Cre (with the Cre allele assessed using the following PCR primers: 5’- TGCCACGACCAAGTGACAGCAATG-3’ and 5’- AGAGACGGAAATCCATCGCTC-3’). *Npnt+/-* founders were then bred to produce *Npnt-/-* mice. Animals were housed in a specific pathogen-free facility with a 12-h light-dark cycle and had free access to food and water. All animal work was approved by the Institutional Animal Care and Use Committees at the University of Wisconsin-Madison and the Medical University of South Carolina and adhered to the ARRIVE guidelines (S1 Checklist).

### LPS acute lung injury model

Both male and female mice derived from 5 breeder cages and multiple litters were used for the study. All mice were administered LPS, with Cre- animals serving as controls. Mice were used at 10–12 weeks of age, with LPS administration and experimental endpoints staggered according to their age across 11 groups rather than by randomization. Twelve Cre- and 14 Cre+ mice were used for day 3 time points and 20 Cre- and 23 Cre+ were used for day 7 time points. Sample sizes were selected to provide a 95% power to detect a 50% difference in injury parameters at a significance level of 0.05. Animals were anesthetized with isoflurane and given *E*. *coli* LPS (Sigma, catalog #L2630) at a dose of 1 mg/kg by oropharyngeal aspiration. Body weight and overall health were monitored daily. For this study, no animals were euthanized for sickness or distress (excessive weight loss, decreased mobility and appetite, a hunched appearance, lack of grooming). Mice were euthanized at the intended time points by isoflurane overdose for collection of bronchoalveolar lavage fluid (BALF) and lung tissue as previously described [23]. The same person (C.L.W.) collected samples at each time point to minimize potential confounders from using multiple groups of mice at different times. Only C.L.W. was aware of the genotypes during the group allocation; individual mice were identified only by a unique number during data acquisition.

### Real-time qPCR

Total RNA was isolated from homogenized lung tissue using the Aurum^™^ Total RNA Mini kit (Bio-Rad, Hercules, CA) in conjunction with DNase treatment as per the manufacturers’ specifications. Total RNA was reverse-transcribed to cDNA using iScript Reverse Transcription SuperMix (Bio-Rad). Real-time PCR was done using an ABI OneStep Plus instrument with ABI TaqMan Gene Expression Assays. Quantification of gene expression was normalized to *B2m* or *Rpl13a* (endogenous controls). Taqman assays include: *B2m* (Mm00437762_m1), *Ccl2* (Mm00441242_m1), *Egfl6* (Mm00469452_m1), *Il10* (Mm01288386_m1), *Itga8* (Mm01324958_m1), *Npnt* (Mm00473794_m1 for analysis of overall expression, Mm00473783_m1 for analysis of knockdown), *Rpl13a* (Mm01612986_gH), *Tgfb* (Mm00441724_m1), and *Tnfa* (Mm00443258_m1). Relative expression was determined by the 2^-ΔΔCT^ method.

### Histology

For measurement of mean linear intercept (MLI), lung sections were stained with hematoxylin and eosin and, for each subject, 5 images of the parenchyma were taken at 200X using a Nikon Eclipse Ti-U microscope. MLI values were obtained using Image J v1.53 and the “Measure MLI” plug-in developed by Crowley et al. [24].

### Immunofluorescence for NPNT

Formaldehyde-fixed paraffin-embedded sections were steamed in IHC-Tek Epitope Retrieval Solution (IHCWorld) for 40 minutes, then blocked with Tris-buffered saline containing 0.05% Tween-20 (TBST) and 5% donkey serum. Sections were incubated overnight at 4°C with a goat anti-mouse NPNT polyclonal (R&D, catalog number AF4298) or IgG isotype control (Jackson Immunoresearch, catalog number 005-000-003) at 2 μg/ml in TBST containing 1% BSA. Staining was visualized using Alexa Fluor555-conjugated donkey anti-goat (Invitrogen) antibody at 4 μg/ml and images were captured at an equivalent laser intensity with a Nikon Eclipse Ti-U epifluorescence microscope.

### Isolation and chemotaxis of bone marrow-derived macrophages (BMDMs)

Bone marrow was extracted from WT and NPNT KO mice to generate BMDMs by culturing the cells in RPMI-1640 supplemented with 10% FBS, 20% L929-conditioned medium, and penicillin/streptomycin for 7–10 days at 37°C and 5% CO_2_. Transwell inserts with 5-μm pore size polycarbonate membranes were seeded with 1.5 x 10^5^ cells per insert in 24-well tissue culture plates (Corning). Following cell attachment at 37°C for 1 h, media in the inserts were replaced with serum-free media. Serum-free media (as a negative control) or LPS BALF diluted in serum-free media (1:25) from Cre- and Cre+ mice were added to wells to assess chemotaxis. Cells were allowed to migrate to the underside of the polycarbonate membrane for 24 h at 37°C, after which cells remaining in the insert were gently removed with cotton-tipped swabs. Migrated cells were fixed and stained by submerging inserts in 70% ethanol for 10 min at room temperature, drying them completely, and incubating with 0.2% crystal violet in 20% methanol for 30 min at room temperature. Membranes were washed with water and dried before imaging under light microscopy at 20X magnification using 5 predetermined fields. The number of counted cells was averaged and expressed as number of cells per field.

### BALF cytokine measurement by Luminex

Cytokine levels were measured using the ProcartaPlex 12-Plex Assay for Mouse (ThermoFisher) (GROα, IL-1^®^, IL-6, IL-10, IL-12p70, IP-10, MCP-1, MIP-1α, MIP-1^®^, MIP-2α, RANTES, and TNF-α) with the Luminex^™^ 200^™^ Instrument System (ThermoFisher) located at the University of Washington Histology and Imaging Core.

### Statistics

For normally distributed values, data are reported as mean ± SEM and Student’s t test was used to analyze two groups and one-way ANOVA was used for three or more groups. Non-parametric data are reported as median with 95% confidence interval and were assessed using either the Mann-Whitney test for two groups or the Kruskal-Wallis test, with Dunn’s multiple comparisons post-hoc test, for three or more groups. A *p* value less than 0.05 was considered significant. Statistical calculations were performed using GraphPad Prism 9.3.1.

## Results

### Efficient ablation of NPNT postnatally using the tamoxifen-inducible ROSA-Cre model

**Fig 1:**
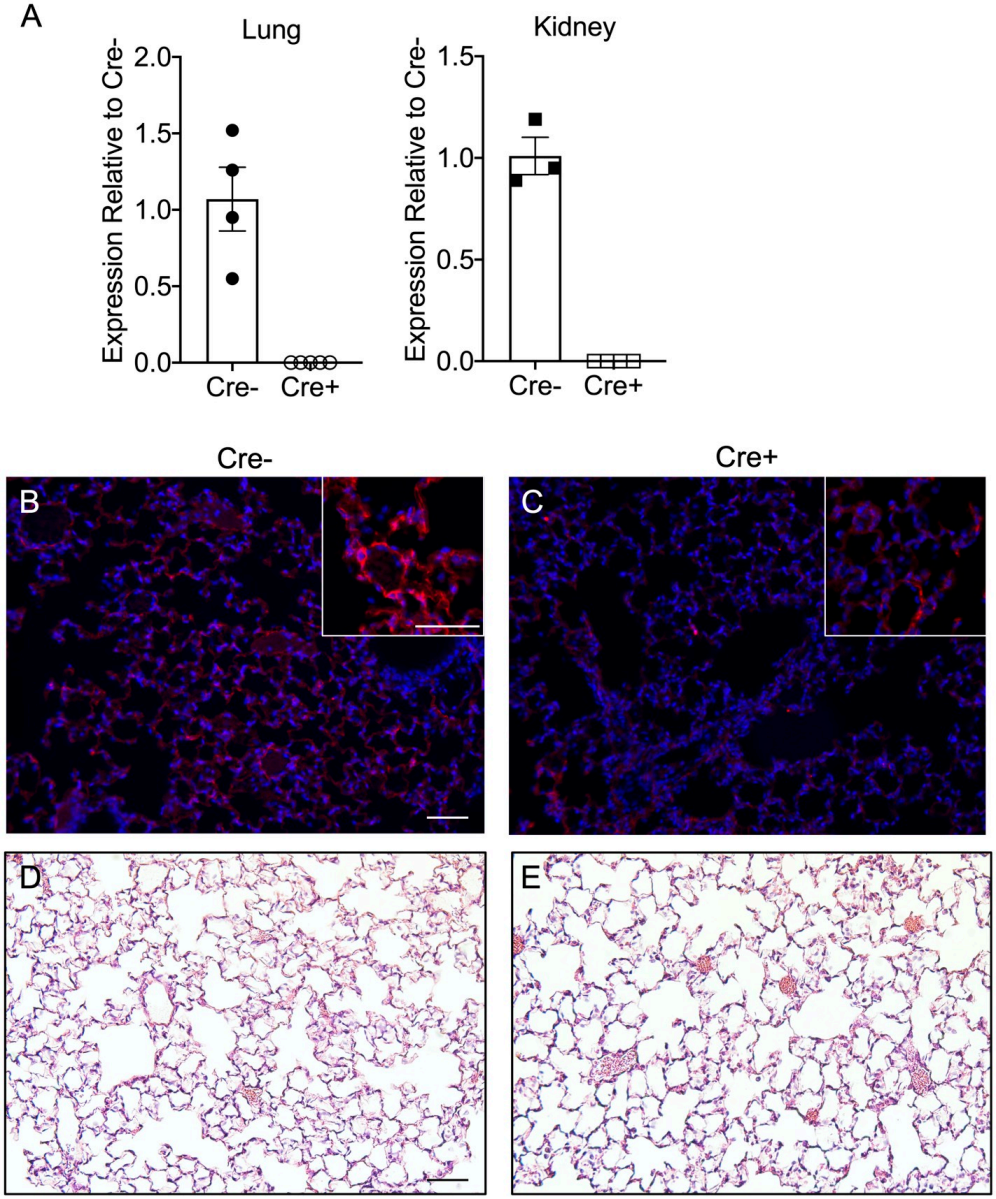
Efficient knockdown of NPNT expression in ROSA-CreER/+;Npnt-Flox/Flox mice after tamoxifen treatment. (A) qPCR analysis of *Npnt* mRNA levels in lung and kidneys from Cre- and Cre+ mice after 4 weeks on the tamoxifen diet followed by normal chow. Expression is shown relative to Cre-. Shown are the means (bars) ± SE. Immunofluorescence staining for NPNT (red) in Cre- (B) and Cre+ (C) lungs from mice fed a tamoxifen diet for 4 weeks followed by chow. Nuclei were stained with DAPI. The insets show the localization of NPNT in Cre- and reduced staining in Cre+ lung tissue. Representative hematoxylin and eosin staining of sections of lung from uninjured Cre- (D) and Cre+ (E) using the same time course of dietary treatment. Scale bar = 50 μm for all images.

Because mice with global deletion of NPNT have limited survival due to defects in kidney morphogenesis, we leveraged animals with a floxed allele for conditional knock out of NPNT postnatally [9] and crossed them with mice harboring a tamoxifen-inducible Cre recombinase expressed from the ROSA locus. Weaning ROSA-CreERT2/+;Npnt-Flox/Flox (Cre+) and control Npnt-Flox/Flox (Cre-) pups onto a tamoxifen diet and feeding this diet for 3–4 weeks resulted in efficient deletion of exon 1 from *Npnt* mRNA in the lungs and kidneys of Cre+ mice (Fig 1A). After switching the mice to regular chow and maintaining them on this diet for 3–4 weeks, we assessed NPNT protein levels in the lungs by immunofluorescence: in Cre- mice, the pattern of NPNT immunoreactivity is consistent with localization to the basal laminae in the small airways (Fig 1B), as was shown in the developing mouse lung [4]. NPNT was also prominent in the alveoli (Fig 1B). Cre+ lungs showed substantial diminution of the signal for NPNT, although isolated areas of staining were still evident (Fig 1C). Deletion of NPNT did not overtly affect lung morphology in Cre+ mice as compared to Cre- at baseline (Fig 1D and 1E); we assessed lung structure quantitatively by measuring the mean linear intercept and found that these values were not different between the genotypes (average of 49.89 μm ± 4.17 SE for Cre- and 50.04 μm ± 1.97 SE for Cre+). There were no deleterious effects of NPNT deletion on the health or survival of Cre+ animals.

### LPS-induced lung injury decreases Npnt mRNA but not protein in Cre- controls

**Fig 2:**
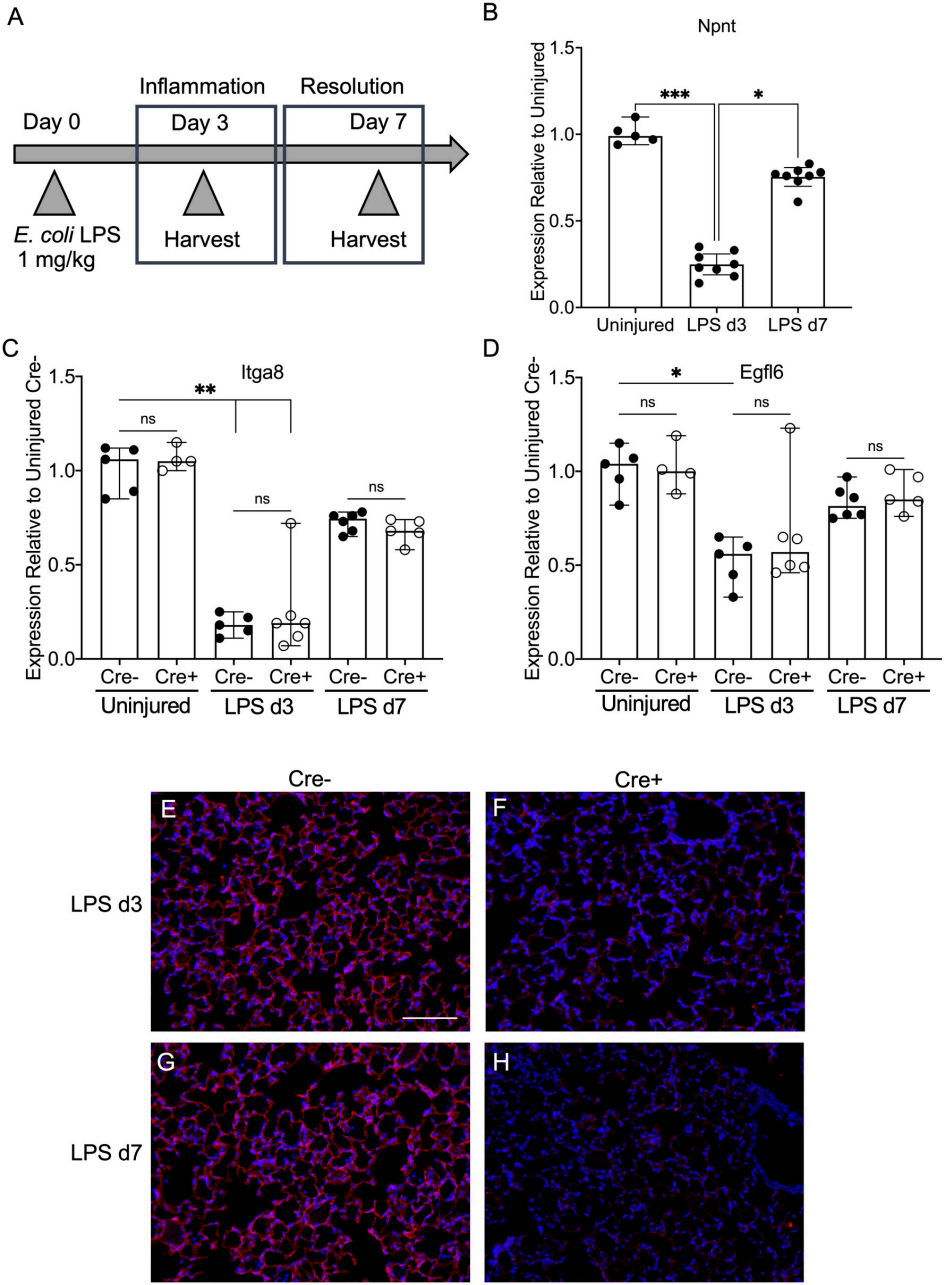
Effect of LPS lung injury on expression of *Npnt* and related genes. (A) Schematic of the time course of LPS injury and sample acquisition. (B) mRNA levels of *Npnt* in lungs from Cre- mice were measured by qPCR and expression calculated relative to uninjured. qPCR was also used to determine if expression of integrin α8 (*Itga8*) (C) or the NPNT homologue EGFL6 (D) is altered by LPS injury of Cre+ lungs. Gene expression was calculated relative to uninjured Cre-. Shown are the medians (bars) with 95% confidence intervals. Data were analyzed using either the Mann-Whitney or Kruskal-Wallis statistical test. *, p < 0.05; **, p < 0.01; ***, p < 0.001; ns, not significant. (E-H) Immunofluorescence for NPNT at day 3 (E and F) and day 7 (G and H) post LPS in Cre- (E and G) and Cre+ (F and H) lung tissue. Nuclei were stained with DAPI (blue). Scale bar = 100 μm.

To examine a potential role for NPNT in lung injury, inflammation, and resolution, Cre+ and Cre- mice were used in the well-established LPS model of acute lung injury (ALI). A sublethal dose of the endotoxin was administered to the lungs via the oropharyngeal route for direct injury of the epithelium. At time points representing the peak of inflammation (day 3) and the resolution phase (day 7), lung tissue and bronchoalveolar lavage fluid (BALF) were obtained and used for analysis as outlined in the schematic (Fig 2A). We first assessed the expression of *Npnt* in Cre- lungs by qPCR during the inflammatory process. We found that *Npnt* mRNA decreased at day 3 relative to uninjured lungs (by an average of 75%), but expression began returning to baseline levels during injury resolution (Fig 2B). This expression pattern is strikingly similar to that observed for the major NPNT receptor, integrin α8 (*Itga8*), which also decreased with LPS by 75–82% (Fig 2C), as was previously demonstrated for LPS-treated mouse fetal lung explants and mesenchymal cells [25]. Deletion of *Npnt* mRNA in lungs from Cre+ mice did not alter the expression levels of integrin α8 relative to Cre- (Fig 2C) during the injury time course. Expression of the NPNT homologue and integrin α8 ligand EGFL6 (or MAEG) [26] was also reduced by LPS, but there were no differences between Cre- and Cre+ (Fig 2D), demonstrating that a compensatory increase in *Egfl6* mRNA does not occur in Cre+ lungs. Although expression of all 3 genes was transiently dampened by LPS at day 3, transcripts were still readily detectable by RT-PCR, with Ct values ranging from 25–28 at this time point (S1 Table).

While we detected a significant decrease in *Npnt* RNA levels with LPS treatment, the rate of protein turnover during inflammation is not known. Therefore, we examined expression levels of NPNT by immunofluorescence. We found that NPNT was still robustly expressed in lungs of Cre- mice after LPS injury (Fig 2E and 2G), in contrast to integrin α8, where both RNA and protein are decreased after LPS stimulation (Fig 2C and [25]). Lungs from Cre+ animals had barely detectable levels of NPNT, especially at day 7 (Fig 2F and 2H).

#### No difference between Cre- and Cre+ in lung injury at day 3 post LPS

**Fig 3:**
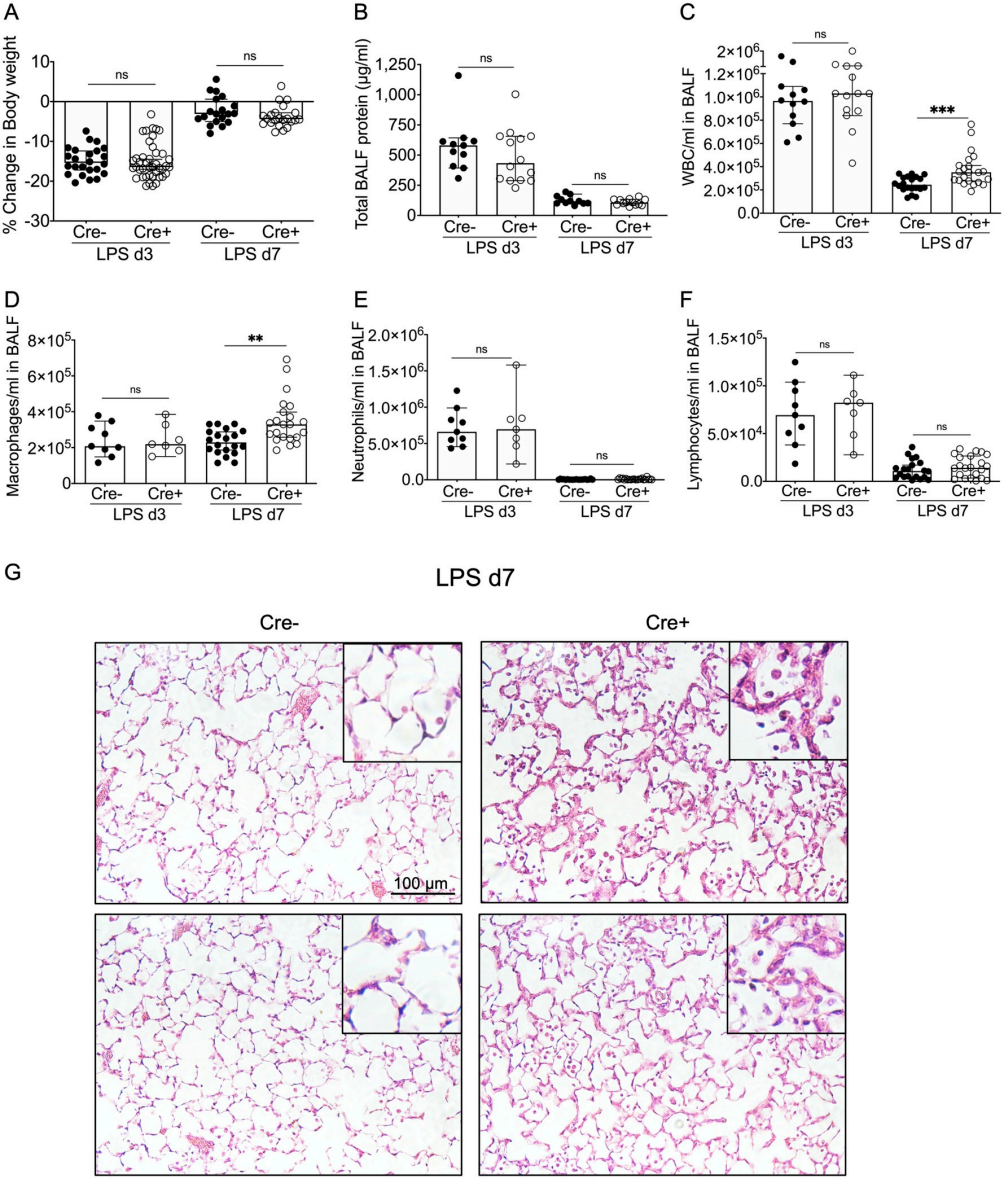
Characterization of lung injury and resolution in the ROSA-CreER;Npnt-Flox/Flox model. Shown are the changes in body weight (A), BALF total protein (B), and total WBC/ml in the BALF (C) at days 3 and 7 post LPS. The body weights of mice used for the day 7 time point were included in the day 3 data. Differential staining of cytospins was done to assess the macrophages (D), neutrophils (E), and lymphocytes (F) in BALF at the two time points. Shown are the medians (bars) with 95% confidence intervals. The Mann-Whitney statistical test was used to compare Cre+ to Cre- values. **, p < 0.01; ***, p < 0.001; ns, not significant. (G) Hematoxylin and eosin staining of sections of lung from 2 representative Cre- and 2 Cre+ mice at day 7 post LPS. Insets depict magnified areas (by 3-fold) of each image. Scale bar = 100 μm.

Next, we evaluated multiple parameters of lung injury and inflammation in LPS-treated Cre- and Cre+ mice. As expected, the mice showed maximum weight loss at day 3 post LPS, but no differences were noted between Cre- and Cre+ animals (Fig 3A). LPS causes a transient rise in alveolar-capillary permeability, resulting in the expected increase in total protein in the BALF at day 3, with levels approaching baseline by day 7 (Fig 3B). However, there was no significant difference in protein concentration in BALF from Cre+ as compared to Cre- at day 3 after LPS administration (Fig 3B). The concentration of WBCs was also similar between the genotypes at this time point (Fig 3C). Neutrophils predominated in the BALF of both Cre- and Cre+ lungs, with no difference in the concentration of these cells as well as that of macrophages and lymphocytes. These results indicate that the initial injury response to LPS is not overtly different without NPNT. No overt differences in immunostaining for either collagen IV or laminin were evident between Cre+ and Cre- lungs (S1 Fig).

### Cre+ mice have a persistent macrophage infiltrate at day 7 post LPS

In the resolution phase of lung injury (day 7 post LPS), Cre+ mice had body weight recovery similar to Cre- (Fig 3A) and re-establishment of barrier function, as assessed by protein concentration in the BALF (Fig 3B). However, compared to Cre-, Cre+ lungs had a significantly higher number of nucleated cells in the BALF at day 7 (Fig 3C), due to an increased number of macrophages (Fig 3D), with no differences in neutrophils (Fig 3E) or lymphocytes (Fig 3F). Histologically, increased macrophages were clearly present in the airspace of Cre+ lungs at day 7; in addition, alveolar septal thickening, a key feature of lung injury, was still evident (Fig 3G). By contrast, Cre- lungs showed essentially normal architecture and minimal macrophages (Fig 3G). As at day 3, we found no differences in immunoreactivity for collagen IV and laminin between the genotypes at this time point (S1 Fig).

**Fig 4:**
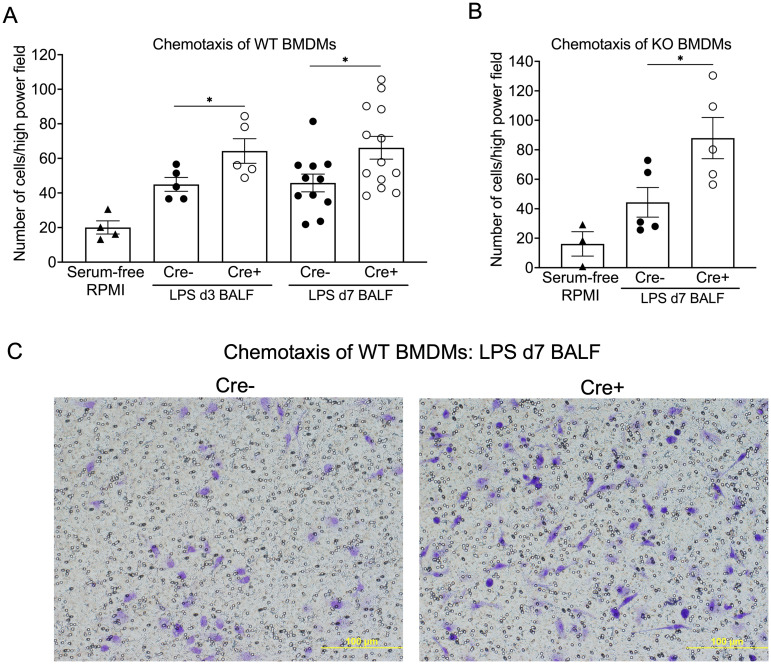
BALF from Cre+ mice promotes chemotaxis of WT and NPNT KO BMDMs. WT (A) or KO (B) cells were seeded into transwells containing diluted d3 or d7 post-LPS BALF from either Cre- or Cre+ mice in the bottom chambers. Serum-free RPMI-1640 medium was used as a control. Shown are the means (bars) ± SE. Data were analyzed using an unpaired Student’s t test to compare Cre+ to Cre- values at each time point. *, p < 0.05. (C) Shown are representative images of WT BMDMs (blue) that have migrated to the other side of the filter in response to BALF recovered from Cre- and Cre+ mice at day 7 after LPS.

To investigate potential mechanisms for the increased number of macrophages in Cre+ lungs in the resolution phase, we isolated bone marrow-derived macrophages (BMDMs) from WT and NPNT KO mice and assessed their chemotactic response to BALF from day 3 and day 7 post-LPS lungs. We found that a greater number of cells migrated in response to Cre+ BALF than to Cre- at both time points (Fig 4A and 4C), indicating that the Cre+ BALF had increased chemotactic activity for macrophages. In response to day 7 BALF, chemotaxis of BMDMs from NPNT KO mice was similar to WT, indicating that the migratory activity was cell extrinsic (Fig 4B). We further demonstrated that the increase in macrophage numbers was not due to proliferation, as neither Cre+ nor Cre- d7 BALF promoted the growth of WT BMDMs under serum-free conditions (S2 Fig).

### No consistent difference between Cre- and Cre+ lungs in expression of chemokines/cytokines

**Fig 5:**
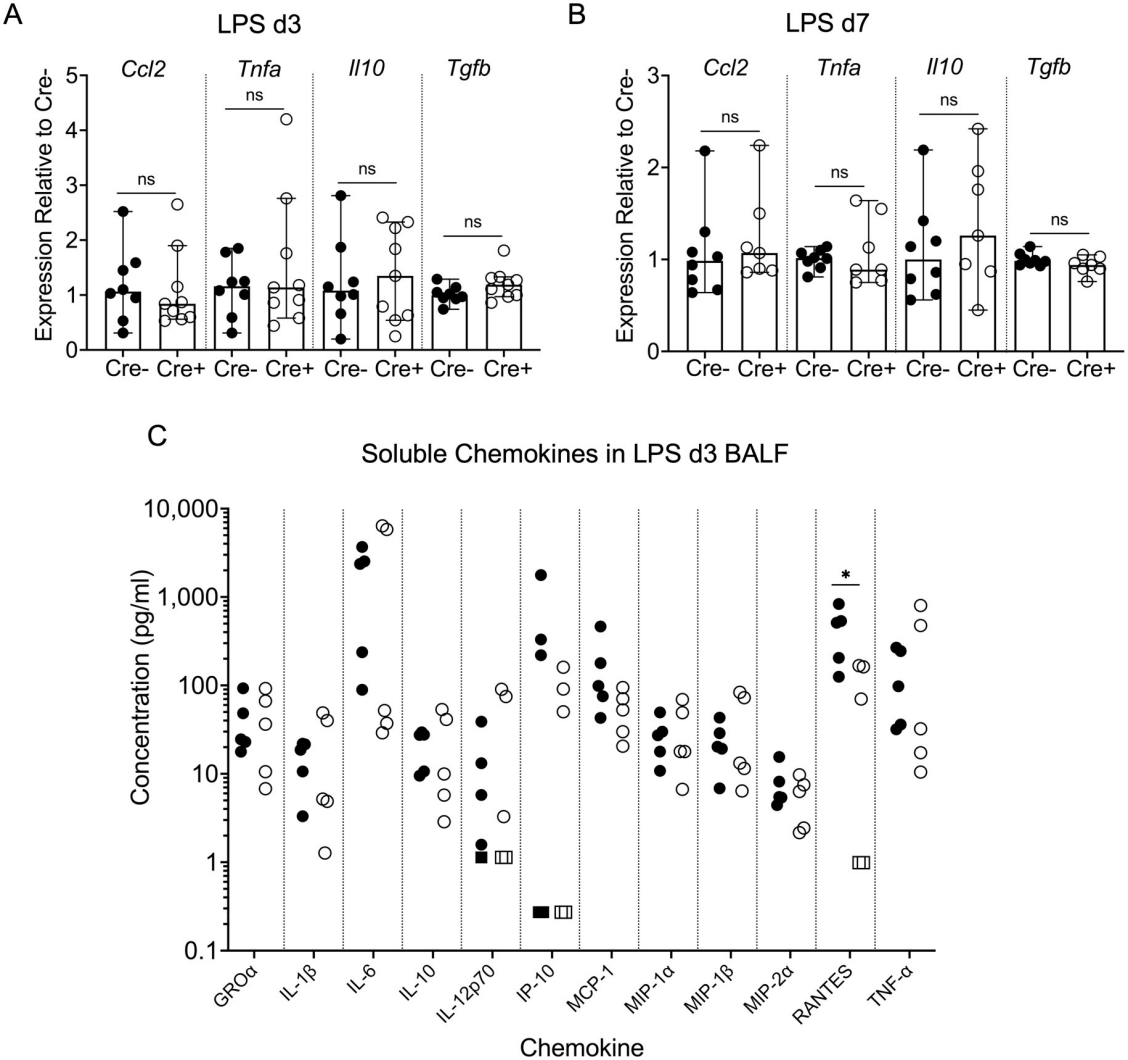
Elaboration of inflammatory mediators during LPS injury and resolution. qPCR of both proinflammatory (*Ccl2* and *Tnfa*) and anti-inflammatory genes (*Il10* and *Tgfb*) was performed on lung left lobe RNA isolated from Cre- and Cre+ animals at days 3 (A) and 7 (B) after LPS administration. Expression levels in Cre+ lung RNA samples were calculated relative to Cre- for each gene. Shown are the medians (bars) with 95% confidence intervals. The Mann-Whitney statistical test was used to compare Cre+ to Cre- values. ns, not significant. (C) Chemokine levels in Cre- (filled symbols) and Cre+ (open symbols) BALF at day 3 after LPS were measured by a Luminex assay. Values depicted by the square symbols were at or below the limit of detection for that analyte. Cre+ values were compared to Cre- by Mann-Whitney tests.

We then analyzed the elaboration of key pro- and anti-inflammatory mediators (Fig 5) to determine if differences in these factors could account for the enhanced chemotactic activity of the Cre+ BALF at day 7 post LPS. We determined that Cre+ lung tissue did not differ from Cre- in expression of *Ccl2* (MCP-1), an important chemokine in monocyte recruitment, at either day 3 or day 7 after LPS treatment (Fig 5A and 5B). *Tnfa* expression was not different at either time point, indicating that both Cre- and Cre+ responded equivalently to LPS, which induces TNF-α. Expression of the anti-inflammatory mediators *Il10* and *Tgfb* was similar between the genotypes as well (Fig 5A and 5B). Soluble levels of chemokines in the BALF were determined using a multiplex panel. There were no consistent differences in chemokine concentrations in Cre- and Cre+ BALF at day 3 after LPS, except for a statistically significant lower level of RANTES (CCL5) in the Cre+ BALF (Fig 5C). By day 7, soluble chemokines were either undetectable or were present at low levels that did not differ between Cre- and Cre+ BALF (S2 Table).

## Discussion

Originally identified as a BM protein required for kidney morphogenesis in mice, NPNT is prominently expressed in the mouse lung under baseline conditions [19], as is the primary receptor for NPNT, integrin α8β1 [25, 27, 28]. Given the importance of the BM in lung homeostasis and in regulating lung injury, we asked if the absence of NPNT would affect baseline lung morphology, the initial inflammatory response, or the resolution phase of lung injury.

Our findings showed that postnatal deletion of NPNT, using a tamoxifen-inducible ROSA-Cre model, does not overtly alter alveolar architecture (Fig 1) or lung permeability at baseline. The half-life of NPNT in the alveolar BM in the absence of injury is not known. However, our immunofluorescence data suggest that in our model, a period of 6 to 8 weeks of tamoxifen exposure and wash-out is sufficient for turnover of the majority of the pre-existing NPNT in lung BMs. How the level of NPNT protein in BMs is regulated under homeostatic conditions remains to be determined. Our data show that NPNT is dispensable for normal barrier function and homeostasis in the postnatal lung. Because the NPNT homologue EGFL6 is also expressed in the lung and localizes to BMs in the alveoli [4], EGFL6 may compensate for the absence of NPNT, as it does in development of hair follicles in *Npnt*-/- mice [29].

We then used the LPS model of ALI to determine if perturbation of the air-blood barrier by injury and inflammation affects expression of NPNT. We showed that similar to integrin α8, *Npnt* transcripts are downregulated by an average of 75% in the lung at the peak of inflammation (day 3 post LPS) but recover close to basal levels by day 7 (Fig 2). There is precedent for regulation of *Npnt* expression by proinflammatory factors, as TNF-α, which is induced by LPS, also suppresses *Npnt* mRNA in a mouse osteoblastic cell line in a time- and dose-dependent manner [30]. In the lung, NPNT is expressed by both epithelial cells and alveolar fibroblasts and is deposited into BMs, whereas α8 is synthesized by and functions in mesenchymal cells. Benjamin et al. showed that both the mRNA and protein of integrin α8 are reduced in mouse fetal lung explants or mesenchymal cells exposed to LPS [25]. These investigators found that expression of other α integrin subunits (α2, α3, α5, and α9) was not significantly affected by LPS, supporting the idea that α8 is a specific target during inflammation. However, we did not observe a decrease in NPNT immunoreactivity in response to LPS (Fig 2). Consistent with our finding, another group employing a dynamic proteomic approach to measure fractional synthesis rates of ECM proteins demonstrated that NPNT protein was not significantly changed during the inflammatory phase of bleomycin-induced lung injury [11]. Regardless, an inflammation-associated reduction in integrin α8 protein, the major receptor for NPNT, would likely impact signaling through this ligand-receptor pair. Transcriptional expression of EGFL6, also a ligand of integrin α8 [26], was similarly reduced by LPS at day 3, but not as dramatically (average of 48%).

To test the requirement for NPNT in acute injury and its resolution, we used the LPS model in mice with conditional deletion of NPNT in all cells. NPNT deletion did not augment the initial inflammatory response to LPS-induced injury: Cre+ (NPNT deficient) mice were similar to Cre- in degree of body weight loss, changes in alveolar-capillary permeability, and influx of leukocytes, primarily neutrophils, at day 3 (Fig 3). By day 7 post LPS, when resolution is well underway, neutrophil numbers decreased in both the Cre- and Cre+ BALF, suggesting that this phase initiates normally in the mutants. However, there was a greater number of leukocytes, specifically macrophages, in Cre+ BALF as compared to Cre- at this time point. This was not due to increased permeability of the air-blood barrier in Cre+ lungs, as total protein concentration in the BALF of both Cre- and Cre+ had returned to essentially normal levels by day 7. *In vitro* experiments using WT BMDMs with day 7 BALF confirmed that this increase was not due to promotion of proliferation (S2 Fig); rather, Cre+ BALF at this time point and at day 3 had a greater chemotactic activity for macrophages than Cre- (Fig 4). This activity did not correlate with increased expression or levels of typical macrophage-centric chemokines, such as MCP-1 (CCL2), in the lung tissue or BALF (Fig 5). In fact, BALF from Cre+ lungs had a significantly lower concentration of RANTES (CCL5) at day 3, but this difference did not persist to day 7. Both genotypes at day 7 post LPS had low or undetectable levels of chemokines (S2 Table). There may be differences in other chemokines or soluble factors that influence leukocyte trafficking, such as matrikines (proteolytic fragments of ECM proteins), that were not measured in our assays. Despite the enhanced chemotactic activity in day 3 BALF from Cre+ lungs, we did not observe a higher number of macrophages in the BALF relative to Cre- at this time point. It is possible that additional time was required for the chemotactic factor(s) to influence the macrophage response *in vivo*. In addition, *in vitro*-differentiated macrophages may differ in their sensitivity and/or behavior towards chemotactic factors than *in vivo* activated cells.

Another possible contributing factor to explain the increased macrophages in the airspace of Cre+ lungs is that absence of NPNT may alter the manner in which these cells interact with BM components. BMDMs do not express significant levels of NPNT relative to lung fibroblasts (S3 Table), and alveolar macrophages do not adhere efficiently to recombinant NPNT (S3 Fig). Although barrier function appeared to be re-established in Cre+ lungs by day 7, based on the return of total protein levels in the BALF to that observed in uninjured lungs, the alveolar wall was not completely normal as compared to Cre- (Fig 3). We found no differences in immunoreactivity for collagen IV and laminin in Cre+ and Cre- lungs with LPS treatment (S1 Fig), indicating that lack of NPNT did not compromise the structural integrity of the BMs. This result is consistent with a report showing that immunolocalization of these BM structural proteins is unaffected in the NPNT KO kidney during development [9]. NPNT may play a role in signaling pathways in lung injury, a possibility that should be explored in future studies. There is precedent for NPNT-α8 signaling in the embryonic kidney, as both proteins are required for mesenchymal expression of glial cell line-derived neurotrophic factor (GDNF) [9]. GDNF is a member of the TGF^®^ superfamily and is necessary for ureteric bud invasion of and branching in the metanephric mesenchyme [31]. BMs in the lung and kidney are remarkably similar in their composition and in their production of matrikines with disease (reviewed in [32]). Because expression of NPNT is efficiently ablated in the kidney, and likely other organs, in the ROSA-CreER model, we cannot rule out the possibility that extrapulmonary deletion of NPNT contributes to the differences observed in the injured lungs of Cre+ mice.

What effect would the increased number of macrophages have on ALI? Macrophages infiltrate the lung during both the induction and resolution phases of ALI, with heterogeneous populations characterizing each phase (reviewed in [33]). Early in ALI, macrophages recruited into the alveolar space are primarily proinflammatory, although a subpopulation of cells with an anti-inflammatory phenotype (high in IL-10 expression) was identified in a *Pseudomonas aeruginosa* pneumonia model [34]. In endotoxin-induced ALI, recruited monocytes replace resident alveolar macrophages, which undergo apoptosis; by day 7, the macrophage population consists of ~50% resident and 50% recruited cells, with recruited cells gradually making up the majority of alveolar macrophages after several months [35]. Our *in vitro* chemotaxis results suggest that this ratio may be skewed toward more recruited than resident macrophages in the NPNT-deficient lung at day 7 post LPS, although this requires formal testing. During resolution, pulmonary macrophages undergo repolarization and assume a reparative gene expression signature [34]. Increased recruitment or retention of macrophages in Cre+ lungs during resolution could conceivably prolong this phase. In addition, the cells may be less efficient in promoting reparative processes in the NPNT-deficient setting. Further work is needed to characterize the phenotype of the alveolar macrophages during ALI in the absence of NPNT.

In summary, we have shown that postnatal deletion of NPNT does not affect the lungs at baseline and does not change the inflammatory response and initiation of repair after LPS challenge. Instead, we speculate that NPNT may be required for completion of the resolution phase. That NPNT is important in injury repair is supported by a recent analysis of BM proteomes in bleomycin-induced lung injury and fibrosis, where NPNT was one of the BM proteins that is regulated in the remodeling and resolution phases [12]. In future studies, evaluation of other models of lung injury, as well as investigation of signaling pathways that may be affected by NPNT deletion, will be instrumental in further delineating the role of NPNT in lung injury and repair.

## Supporting information

S1 Fig No differences in immunofluorescence for collagen IV or laminin in LPS-challenged Cre- and Cre+ lungs. Lung sections from Cre- and Cre+ were stained for collagen IV or laminin at day 3 and day 7 post LPS. Sections were processed as described in the Materials and Methods, using rabbit polyclonals against collagen IV (Millipore Sigma, #AB756P) and laminin (Novus, #NB300-144). Nuclei were stained with DAPI (blue). Scale bar = 50 μm.
(PDF)

S2 Fig LPS day 7 BALF lungs does not promote proliferation of bone marrow-derived macrophages (BMDMs). WT BMDMs were isolated and differentiated as described in the Materials and Methods. Cells were seeded at a density of 1 x 10^4^ cells per well in a black 96-well plate with clear flat bottoms. Cells were exposed to treatment media for 24 h at 37°C. Proliferation was assayed using the CyQuant Cell Proliferation Kit (ThermoFisher, #C7026). Shown are the means (bars) ± SE. Data were analyzed using a one-way ANOVA with Tukey’s multiple comparisons test. ns, not significant.
(PDF)

S3 Fig Alveolar macrophages adhere less efficiently to NPNT than to fibronectin (FN). Alveolar macrophages were harvested from WT mice by BAL, and 60,000 cells in RPMI were added to wells coated with either rat plasma FN (Sigma, #F0635) or recombinant mouse NPNT (R&D, #4298-NP), both at 10 μg/ml. After 2 h, unattached cells were removed and adherent cells were fixed and stained with 1% paraformaldehyde/0.5% crystal violet. Images are representative of 3 independent experiments.
(PDF)

S1 Table Average Ct values from RT-PCR of total lung RNAs. (PDF)

S2 Table Luminex analysis of chemokines in BALF at day 7 post LPS (pg/ml). (PDF)

S3 Table Ct values from RT-PCR of BMDM and fibroblast RNAs. BMDMs were harvested and prepared from WT mice as described in the Materials and Methods. WT mouse lung fibroblasts were obtained by collagenase digestion and cultured in DMEM containing 10% FBS. RNA was isolated and analyzed as described in the Materials and Methods.
(PDF)

S1 Checklist The ARRIVE guidelines 2.0. (PDF)
