# Supplementary material for: Endotoxin-induced acute lung injury in mice with postnatal deletion of nephronectin
Source: PLoS One. 2022 May 12;17(5):e0268398. doi: 10.1371/journal.pone.0268398 (PMC9097991; doi:10.1371/journal.pone.0268398)
Supplement: S1 Fig — Lung sections from Cre- and Cre+ were stained for collagen IV or laminin at day 3 and day 7 post LPS. Sections were processed as described in the Materials and Methods, using rabbit polyclonals against collagen IV (Millipore Sigma, #AB756P) and laminin (Novus, #NB300-144). Nuclei were stained with DAPI (blue). Scale bar = 50 μm. (PDF) [file pone.0268398.s001.pdf]

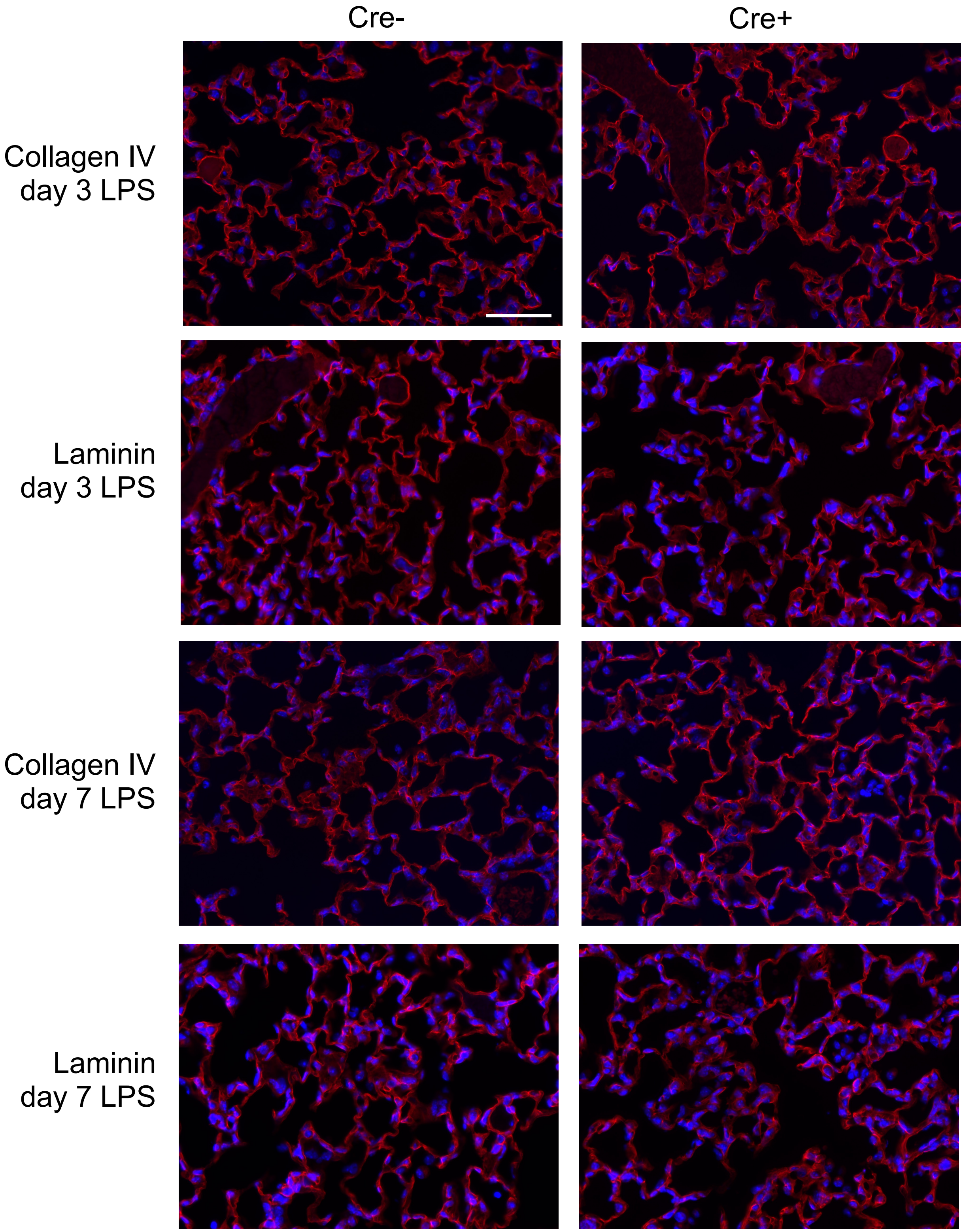

S1 Fig. No differences in immunofluorescence for collagen IV or laminin in LPS-challenged Cre- and Cre+ lungs. Lung sections from Cre- and Cre+ were stained for collagen IV or laminin at day 3 and day 7 post LPS. Sections were processed as described in the Materials and Methods, using rabbit polyclonals against collagen IV (Milipore Sigma, #AB756P) and laminin (Novus, #NB300-144). Nuclei were stained with DAPI (blue). Scale bar = 50 μm.
