# Supplementary material for: Endotoxin-induced acute lung injury in mice with postnatal deletion of nephronectin
Source: PLoS One. 2022 May 12;17(5):e0268398. doi: 10.1371/journal.pone.0268398 (PMC9097991; doi:10.1371/journal.pone.0268398)
Supplement: S2 Fig — WT BMDMs were isolated and differentiated as described in the Materials and Methods. Cells were seeded at a density of 1 x 10^4^ cells per well in a black 96-well plate with clear flat bottoms. Cells were exposed to treatment media for 24 h at 37°C. Proliferation was assayed using the CyQuant Cell Proliferation Kit (ThermoFisher, #C7026). Shown are the means (bars) ± SE. Data were analyzed using a one-way ANOVA with Tukey’s multiple comparisons test. ns, not significant. (PDF) [file pone.0268398.s002.pdf]

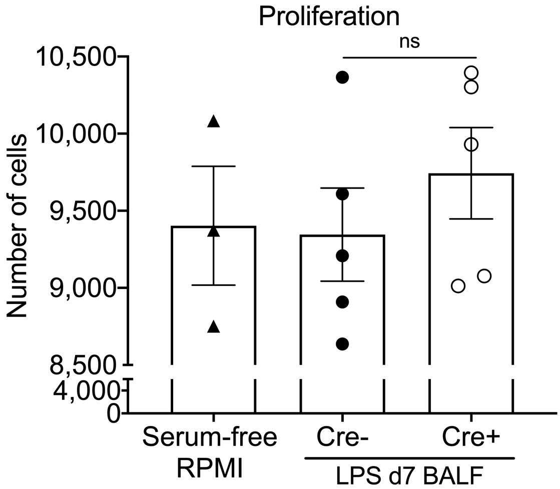

S2 Fig. LPS day 7 BALF lungs does not promote proliferation of bone marrow-derived macrophages (BMDMs). WT BMDMs were isolated and differentiated as described in the Materials and Methods. Cells were seeded at a density of 1 x 10^4 cells per well in a black 96-well plate with clear flat bottoms. Cells were exposed to treatment media for 24 h at 37°C. Proliferation was assayed using the CyQuant Cell Proliferation Kit (Thermofisher, #C7026). Shown are the means (bars) ± SE. Data were analyzed using a one-way ANOVA with Tukey’s multiple comparisons test. ns, not significant.
