# Supplementary material for: Endotoxin-induced acute lung injury in mice with postnatal deletion of nephronectin
Source: PLoS One. 2022 May 12;17(5):e0268398. doi: 10.1371/journal.pone.0268398 (PMC9097991; doi:10.1371/journal.pone.0268398)
Supplement: S3 Fig — Alveolar macrophages were harvested from WT mice by BAL, and 60,000 cells in RPMI were added to wells coated with either rat plasma FN (Sigma, #F0635) or recombinant mouse NPNT (R&D, #4298-NP), both at 10 μg/ml. After 2 h, unattached cells were removed and adherent cells were fixed and stained with 1% paraformaldehyde/0.5% crystal violet. Images are representative of 3 independent experiments. (PDF) [file pone.0268398.s003.pdf]

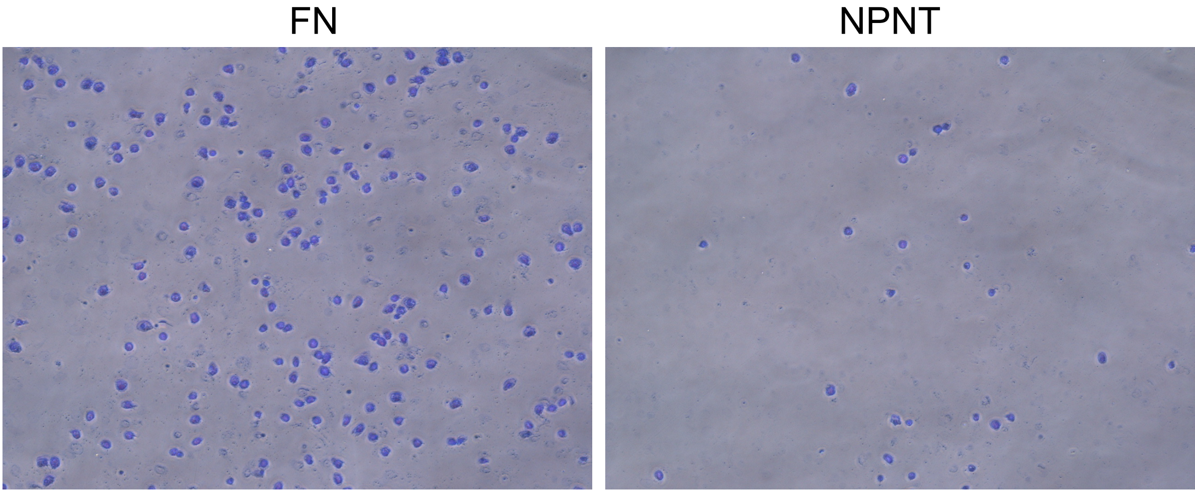

S3 Fig. Alveolar macrophages adhere less efficiently to NPNT than to fibronectin (FN). Alveolar macrophages were harvested from WT mice by BAL, and 60,000 cells in RPMI were added to wells coated with either rat plasma FN (Sigma, #F0635) or recombinant mouse NPNT (R&D, #4298-NP), both at 10 μg/ml. After 2 h, unattached cells were removed and adherent cells were fixed and stained with 1% paraformaldehyde/0.5% crystal violet. Images are representative of 3 independent experiments.
