# Supplementary material for: Endotoxin-induced acute lung injury in mice with postnatal deletion of nephronectin
Source: PLoS One. 2022 May 12;17(5):e0268398. doi: 10.1371/journal.pone.0268398 (PMC9097991; doi:10.1371/journal.pone.0268398)
Supplement: S1 Table — (PDF) [file pone.0268398.s004.pdf]

S1 Table. Average Ct values from RT-PCR of total lung RNAs

Samples	*Rpl13a*	*Npnt*	*Rpl13a*	*Egfl6*	*Rpl13a*	*Itga8*
Baseline	21.71	23.34	22.41	24.50	21.56	24.64
d3 LPS	21.91	25.59	22.55	25.48	21.73	27.30
d7 LPS	21.21	23.25	21.86	24.19	20.98	24.61
