# Supplementary material for: Endotoxin-induced acute lung injury in mice with postnatal deletion of nephronectin
Source: PLoS One. 2022 May 12;17(5):e0268398. doi: 10.1371/journal.pone.0268398 (PMC9097991; doi:10.1371/journal.pone.0268398)
Supplement: S2 Table — (PDF) [file pone.0268398.s005.pdf]

S2 Table. Luminex analysis of chemokines in BALF at day 7 post LPS (pg/ml)

Sample	GROα	IL-1β	IL-6	IL-10	IL-12p70	IP-10	MCP-1	MIP-1α	MIP-1β	MIP-2α	RANTES	TNF-α
Cre- 1	8.32	*	3.84	9.44	*	1.74	7.53	0.60	0.54	1.37	4.90	4.08
Cre- 2	*	*	*	*	*	*	*	*	*	*	*	*
Cre- 3	*	*	*	5.02	*	1.29	*	*	*	*	5.55	*
Cre- 4	*	*	*	*	*	*	*	*	*	*	3.16	*
Cre- 5	*	*	*	6.39	*	1.20	*	*	*	2.74	3.52	4.31
Cre+ 1	5.80	*	3.84	14.37	*	1.72	6.15	0.75	0.66	1.80	4.67	4.76
Cre+ 2	6.91	*	4.64	11.94	*	11.46	5.08	0.46	0.59	1.05	4.24	3.39
Cre+ 3	*	*	*	*	*	0.97	*	*	*	*	*	*
Cre+ 4	7.54	*	4.24	15.16	*	1.88	6.15	0.50	0.61	1.52	4.73	3.27
Cre+ 5	*	*	*	4.07	*	1.73	*	*	*	*	2.76	*

*, values at or below minimum detection level
