# Supplementary material for: Endotoxin-induced acute lung injury in mice with postnatal deletion of nephronectin
Source: PLoS One. 2022 May 12;17(5):e0268398. doi: 10.1371/journal.pone.0268398 (PMC9097991; doi:10.1371/journal.pone.0268398)
Supplement: S3 Table — BMDMs were harvested and prepared from WT mice as described in the Materials and Methods. WT mouse lung fibroblasts were obtained by collagenase digestion and cultured in DMEM containing 10% FBS. RNA was isolated and analyzed as described in the Materials and Methods. (PDF) [file pone.0268398.s006.pdf]

S3 Table. Ct values from RT-PCR of BMDM and fibroblast RNAs

	Lung fibroblasts		BMDMs	
Sample	*B2m*	*Npnt*	*B2m*	*Npnt*
Mouse #1	19.16	25.53	20.29	33.08
Mouse #2	18.91	22.85	20.18	34.37
Mouse #3	20.59	24.76	20.17	33.44

BMDMs were harvested and prepared from WT mice as described in the Materials and Methods. WT mouse lung fibroblasts were obtained by collagenase digestion and cultured in DMEM containing 10% FBS. RNA was isolated and analyzed as described in the Materials and Methods.
